# A Systematic Review and Meta-Analysis of Interventions for Actinic Keratosis from Post-Marketing Surveillance Trials

**DOI:** 10.3390/jcm9072253

**Published:** 2020-07-15

**Authors:** Theresa Steeb, Anja Wessely, Matthias Harlaß, Franz Heppt, Elias A. T. Koch, Ulrike Leiter, Claus Garbe, Oliver Schöffski, Carola Berking, Markus V. Heppt

**Affiliations:** 1 Department of Dermatology, Universitätsklinikum Erlangen, Friedrich-Alexander-University Erlangen-Nürnberg (FAU), 91054 Erlangen, Germany; Theresa.Steeb@uk-erlangen.de (T.S.); Anja.Wessely@uk-erlangen.de (A.W.); Franz.Heppt@uk-erlangen.de (F.H.); Elias.Koch@uk-erlangen.de (E.A.T.K.); Carola.Berking@uk-erlangen.de (C.B.); 2 Comprehensive Cancer Center Erlangen-European Metropolitan Area of Nuremberg (CCC ER-EMN), 91054 Erlangen, Germany; 3 Faculty of Health, Medicine and Life Sciences, Maastricht University, 6211 LK Maastricht, The Netherlands; matthias.harlass@t-online.de; 4 Department of Dermatology, Eberhard Karls University of Tübingen, 72076 Tübingen, Germany; ulrike.leiter@med.uni-tuebingen.de (U.L.); claus.garbe@med.uni-tuebingen.de (C.G.); 5 School of Business, Economics and Society, Friedrich-Alexander-University Erlangen-Nürnberg (FAU), 90403 Nürnberg, Germany; oliver.schoeffski@fau.de

**Keywords:** actinic keratosis, phase IV trial, post-marketing surveillance trial, real-world evidence, long-term results, diclofenac, meta-analysis, systematic review, cryosurgery, imiquimod, photodynamic therapy, ingenol mebutate, 5-fluorouracil

## Abstract

Multiple interventions are available for the treatment of actinic keratosis (AK) showing high efficacy in pivotal trials. However, data from post-marketing surveillance studies have received little attention until now. Here, we systematically investigate interventions for AK from post-marketing surveillance trials as a proxy for real-world efficacy and tolerability. A systematic literature search was conducted in Medline, Embase, and CENTRAL. Pertinent trial registers were hand-searched until 25 March 2020. Results were pooled using a random-effects model to calculate pooled proportions and relative risks (RR) or were described qualitatively. Eleven records with a total sample size of *n* = 4109 were included. Three of the studies had an active-controlled design, while seven were single-armed. Participant complete clearance ranged from 23.1% for diclofenac sodium 3% gel to 88.9% for ingenol mebutate 0.05% gel. The lesion-specific clearance rate for photodynamic therapy (PDT) was 74% (95% confidence interval (CI) 56–87%). The recurrence rate was significantly higher for diclofenac sodium 3% in comparison to imiquimod 5% cream (RR 1.10, 95% CI 1.02–1.1.8) and ranged from 10.6% for ingenol mebutate 0.015% gel to 23.5% for PDT. Few patients discontinued the trials due to adverse events. The results from the majority of the post-marketing surveillance studies deviated from those of pivotal trials.

## 1. Introduction

Actinic keratoses (AK) are common precancerous lesions attributable to lifelong exposure to ultraviolet (UV) radiation [1,2]. They belong to the most common skin lesions with a prevalence of up to 60% in Caucasians over the age of 60 years. AK possibly transform into invasive squamous cell carcinoma of the skin (cSCC) [1,2]. Although the transition probability of a single lesion appears to be low, it increases rapidly in the presence of multiple AK, field cancerization, or immunosuppression. Further risk factors for the development of AK, besides advanced age, include male gender and fair skin type. Despite recent advancements in the prognostic classification of AK, pertinent guidelines recommend early and consequent treatment of AK, because it is currently not possible clinically or histologically to exactly delineate whether and which lesions will transform into cSCC [3,4,5,6].

A variety of interventions are available for the treatment of AK in clinical practice, including topical drugs and ablative modalities. Numerous randomized controlled pivotal trials (RCTs) have been published showing that most interventions were superior to placebo in terms of lesion clearance [7]. Thus, multiple interventions are currently authorised for the treatment of AK. After approval of a novel drug or intervention, additional studies are commonly initiated to further investigate efficacy and safety in different settings and subgroups. In particular, post-marketing surveillance trials are undertaken to detect safety signals that have not become evident during phase III trials or to shed light on the long-term efficacy in a real-world setting [8,9,10].

Importantly, data obtained from phase IV trials can differ from those of previous studies and can even lead to withdrawal from the market. However, results from post-marketing surveillance trials of interventions for AK have received little attention until the recent suspension of marketing authorisation for ingenol mebutate (IMB) by the European Medicines Agency (EMA) due to an increased incidence of non-melanoma skin cancer [11]. Hence, this study aimed at performing a systematic review and meta-analysis of phase IV trials in patients with AK as a proxy for real-world efficacy and tolerability.

## 2. Materials and Methods

### 2.1. Protocol and Registration

The protocol for this review was defined a priori and registered online in the PROSPERO international prospective register of systematic reviews (CRD42020146404). This protocol was conducted in accordance with the Preferred Reporting Items for Systematic Reviews and Meta-Analyses (PRISMA) [12] and the Cochrane Handbook for Systematic Reviews [13].

### 2.2. Eligibility Criteria

We included adult patients (≥18 years of age) with a clinical or histopathological diagnosis of AK. Both immunosuppressed and immunocompetent individuals were eligible. The following types of interventions were eligible: surgical approaches (such as excisional biopsies or shave excision), cryosurgery, cryopeeling, ablative lasers (such as erbium: YAG or carbon dioxide laser), IMB 0.015% or 0.05% gel, imiquimod 3.75% or 5% cream, 5-fluorouracil (5-FU) 0.5% or 5% cream, 5-FU 0.5% plus salicylic acid 10% in solution (5-FU/SA), 3% diclofenac in 2.5% hyaluronic acid gel (diclofenac/HA), and photodynamic therapy (PDT) with aminolevulinate (ALA) or its ester methyl-aminolevulinate (MAL) with illumination from light-emitting diodes or natural daylight. Sequential or combination approaches were included. Monotherapy of the interventions mentioned above or placebo served as a comparison in controlled studies. We limited inclusion to study designs that were explicitly designated as “phase IV” or “post-marketing surveillance”, irrespective of randomization. No language restrictions were set.

### 2.3. Types of Outcome Measures

The primary outcomes were: (1) the participant complete clearance, defined as the rate of participants who had all (100%) baseline lesions cleared (dichotomous outcome); (2) the mean lesion complete clearance per patient, defined as the mean proportion (percentage) of cleared lesions (continuous outcome); (3) the lesion-specific clearance, measured as the number of cleared lesions after the end of treatment compared to baseline (dichotomous outcome); (4) recurrence rate, defined as the rate of lesions relapsing after successful clearance (dichotomous outcome). The secondary outcomes were (5) patient satisfaction, i.e., the number of patients rating to be satisfied with the treatment (dichotomous outcome) and (6) the number of patients withdrawing from the study due to adverse events as a proxy of tolerability (dichotomous outcome).

### 2.4. Search Methods for Identification of Studies

We searched the electronic databases Medline, Embase (both via Ovid), and the Cochrane library CENTRAL until 25 March 2020 to identify all relevant records. The search strategies can be obtained from the Supplementary Materials (Table S1). Additionally, we searched the following trial registers for the keywords “actinic keratosis” or “actinic keratoses”: The metaRegister of Controlled Trials (ISRCTN registry www.controlled-trials.com), US National Institutes of Health Ongoing Trials Register (www.clinicaltrials.gov), Australian New Zealand Clinical Trials Registry (www.anzctr.org.au), World Health Organization International Clinical Trials Registry Platform (www.who.int/trialsearch/), EU Clinical Trials Register (www.clinicaltrialsregister.eu/). For ongoing trials and completed trials without data publication, principal investigators or trial sponsors were contacted to obtain preliminary or unpublished data. Reference lists of included records and the European Union electronic Register of Post-Authorisation Studies (EU PAS Register) were screened as well.

### 2.5. Selection of Studies

Two authors (T.S., M.V.H.) independently screened titles and abstracts that were identified in the electronic database searches for eligibility. Trial registers were hand-searched and assessed for eligibility by one author (M.H.). For records that were considered relevant according to title and abstract screening, full-text articles were obtained, and inclusion and exclusion criteria were applied by the same authors. Whenever discrepancies arose, a resolution was achieved by discussion with another independent author (C.B.).

### 2.6. Data Extraction and Management

Information for each included study regarding design, baseline characteristics, intervention, outcomes, and risk of bias were collected and summarized by two authors independently (T.S., M.V.H.) using Microsoft Excel 2010. Pooled proportions for a specific intervention from single-armed studies were calculated with the inverse variance method with the function “metaprop” of the R package “meta” [14]. We used a random-effects model, as clinical and methodological heterogeneity between the studies was likely. Heterogeneity was quantified with the I^2^ statistic. Dichotomous outcomes from controlled trials reporting a specific comparison were expressed as risk ratios (RR) with 95% confidence intervals (CI) and continuous outcomes as mean or median differences (MD) with 95% CI. If meta-analysis for an outcome was not possible, we described the results qualitatively.

### 2.7. Assessment of Risk of Bias and the Certainty of the Body of Evidence

The risk of bias of the included RCTs was assessed independently by two authors (T.S., M.V.H.) by judgement according to the Cochrane Risk of Bias Tool [13]. The remaining studies were evaluated using the Evidence Project risk of bias tool for non-randomized intervention studies [15] and, hence, deviated from the tool we initially planned in the protocol to use for assessing bias in single-arm studies. Discrepancies were thoroughly discussed and resolved with the full texts and Supplementary Materials. If at least 10 RCTs reported a specific comparison, we intended to assess publication bias by creating a funnel plot [13].

## 3. Results

### 3.1. Study Identification

**Figure 1:**
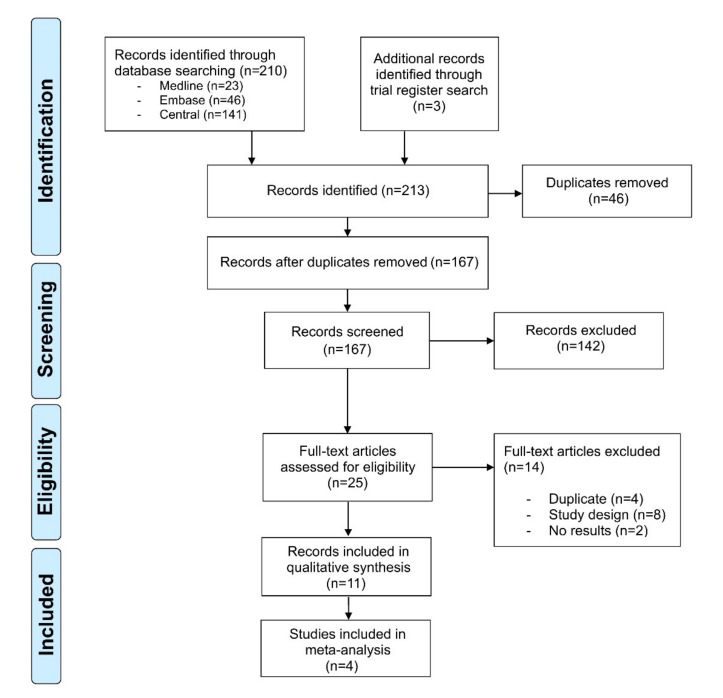
PRISMA flowchart of the study. Selection process for study inclusion in the systematic review and meta-analysis according to the Preferred Reporting Items for Systematic Reviews and Meta-Analysis (PRISMA).

Our literature search identified 213 references. After title and abstract screening and removal of duplicates, 25 records underwent full-text review. Of these, eight records were dismissed, as they did not match the study design, four more duplicates were identified, and two records had not published any results. Hence, 11 records with an overall sample size of *n* = 4109 immunocompetent individuals were included in our review (Figure 1). The diagnosis of AK was made clinically in *n* = 3378, histopathologically in *n* = 556 and both clinically and histopathologically in *n* = 175 patients. Three of the studies had an active-controlled design [16,17,18], while seven were single-armed [19,20,21,22,23,24,25,26] (Table 1). The study by Gollnick et al. presented a pooled analysis of two similar phase IV studies (LEIDA 1 and 2) [17]. All studies assessed AK located on the face or scalp; three studies investigated AK located on the extremities or the trunk as well [20,23,25]. The studies were published between 2006 and 2019. Diclofenac/HA [19,21,22], 5-FU/SA [23,25], and ALA-PDT [24,26] were investigated in two single-armed studies, respectively. However, the two studies investigating PDT differed, as they used either ALA-patch or a topical ALA solution. The remaining studies compared cryosurgery monotherapy with cryosurgery followed by diclofenac/HA [16], imiquimod 5% cream with diclofenac/HA [17], IMB 0.015% gel with diclofenac/HA [18], and one study investigated IMB 0.015% and 0.05% [20]. Due to the differences of the study design and the heterogeneity of the included studies, meta-analysis was performed for two single-armed studies on diclofenac/HA [19,21] and ALA-PDT [24,26], respectively. The results of all other comparisons were reported qualitatively.

### 3.2. Participant Complete Clearance

The participant complete clearance was reported in eight studies. In a single-armed study, one-third of the patients treated with ALA-PDT achieved complete clearance (33.6%) (Table 2 and Table 3) [26]. The participant complete clearance for IMB was 76.6% for lesions located in the face [20] and 88.9% [20] for lesions on the trunk or the extremities. Cryosurgery in combination with diclofenac/HA was more effective in achieving participant complete clearance than cryosurgery monotherapy (RR 1.65, 95% CI 1.33–2.04) [16]. Imiquimod 5% cream and IMB 0.015% gel were both more effective compared to diclofenac/HA in two RCTs (RR 1.47, 95% CI 1.19–1.81; RR 1.92, 95% CI 1.48–2.50) [17,18]. Additional data on diclofenac/HA from two single-armed studies were pooled in a meta-analysis showing participant complete clearance of 35% (95% CI 20–55%) (Figure S1a) [19,21].

### 3.3. Lesion-Specific Complete Clearance

Lesion-specific clearance rates were reported in two single-armed studies investigating ALA-PDT [24,26]. The data were analysed in a meta-analysis revealing 74% clearance (95% CI 56–87%) (Figure S1b).

### 3.4. Recurrence Rate

Three studies reported data for recurrence rates. The recurrence rate was significantly higher for diclofenac/HA in comparison to imiquimod 5% cream (RR 1.10, 95% CI 1.02–1.1.8) [17]. For IMB applied to the face or the scalp, there was a recurrence rate of 10.6%, and for IMB applied to the trunk or the extremities, a recurrence rate of 28.6% was reported [20]. In total, 23.5% of lesions recurred in patients who had undergone ALA-PDT [26].

### 3.5. Mean Lesion Complete Clearance Per Patient

Data on the mean lesion complete clearance per patient were available from six studies. The highest decrease in lesion reduction per patient was reported for 5-FU/SA ranging from 69.7% [25] to 92.3% [23]. Values for diclofenac/HA ranged from 57.5% [18] to 90.5% [21]. For IMB, the decrease ranged from 69.5% [18] to 88.3% [20].

### 3.6. Patient Satisfaction

The number of patients rating to be satisfied with treatment was not reported in any of the studies. Szeimies et al. reported patients to be satisfied with the use of low-dose 5-FU/SA, as they rated this therapy with an average of 2.6 on a scale from 0 to 10 (0 = very satisfied to 10 = not satisfied) [25]. Additionally, in the study by Stockfleth et al., treatment satisfaction was investigated using the Treatment Satisfaction Score for Medication (TSQM), however, no values were reported [18].

### 3.7. Withdrawal Due to Adverse Events (AEs)

The rate of patients who stopped treatment because of AEs was reported in seven studies. Withdrawal due to AEs was higher for patients treated with cryotherapy and diclofenac/HA in comparison to cryotherapy monotherapy (RR 7.20, 95% CI 2.60–20.42) [16]. For diclofenac/HA monotherapy, between 3.9% and 6.1% of patients stopped treatment due to treatment-related AEs [18,22], whereas for IMB, 1.2% to 2.7% of patients withdrew from the study [18,20]. In a direct comparison, fewer patients tended to discontinue treatment due to AEs in the IMB group in comparison to diclofenac/HA (RR 0.45, 95% CI 0.19–1.09) [18]. One study investigating ALA-PDT reported that 1.8% of patients stopped treatment due to treatment-emergent AEs [26]. Withdrawals due to AE occurred in 2% (95% CI 1–3%) of patients treated with 5-FU/SA (Figure S1c) [23,25].

### 3.8. Risk of Bias

**Figure 2:**
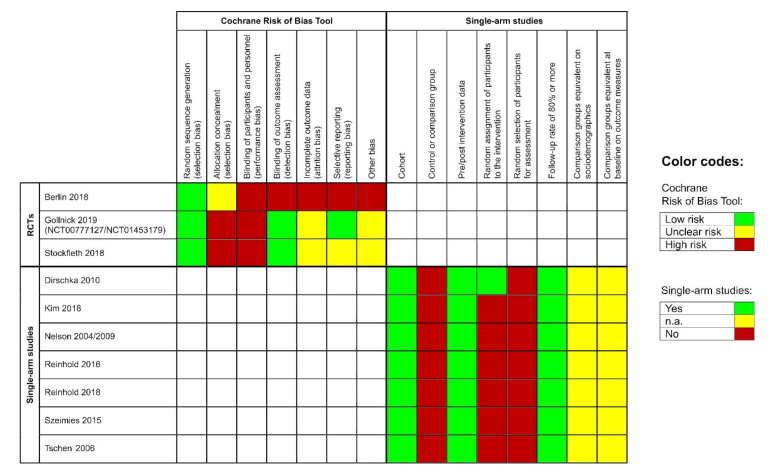
Left side: risk of bias evaluation for each included randomized controlled pivotal trial (RCT); risk of bias summary: review authors’ judgements about each risk of bias item for each included study: ‘+’ = low risk, ‘-’ = high risk, ‘?’ = unclear risk of bias. Right side: risk of bias evaluation of the single-arm studies according to the Evidence Project; n.a. = not assessable.

The included RCTs showed a low risk for bias due to random sequence generation. However, they had a severe risk of performance bias, as none of the participants was blinded. Moreover, allocation concealment was unclear or at high risk for bias. Blinding of the outcome assessor was performed in two of the three studies. Additionally, the studies were at unclear or high risk for attrition bias and the risk for selective reporting varied across all studies (Figure 2, left side). The remaining single-armed studies had a similar risk of bias profile; they showed a high risk for selection bias but a low risk for attrition bias. Pre-and post-intervention data were available for all studies (Figure 2, right side).

## 4. Discussion

A variety of interventions have been authorised for the treatment of AK following phase III RCTs, which have proven their efficacy and safety. Evaluating a drug in a real-world setting helps to complement efficacy data from pre-marketing RCTs and to determine the true safety profile of a drug to support regulatory decision-making [8,10]. Although often neglected, the results are important for patients with AK as well as treating physicians, as was recently demonstrated at the beginning of 2020 when the EMA recommended suspending the use of IMB because a post-marketing analysis revealed higher occurrence of non-melanoma skin cancer with IMB compared to imiquimod 5% cream (3.3% vs. 0.4%). Although the data have not been published to date, the EMA currently recommends suspending the marketing authorisation for IMB in Europe as a measure of precaution [11]. These developments underline the high relevance of post-marketing surveillance trials in the detection of long-term results and safety signals.

Surprisingly, data from post-marketing surveillance studies have mostly been excluded from systematic reviews, meta-analyses, and even guideline recommendations thus far, although they represent an important resource of real-life data that should ultimately be considered in shared decision-making. In this context, our study is the first report to systematically summarize the existing evidence available from such trials for the management of AK. Data on the efficacy outcomes of interest were inconsistently reported. Results for lesion clearance were only available for ALA-PDT, demonstrating clearance of nearly 75% of all lesions, which is somewhat lower compared to previous pivotal trials [4]. Instead, the outcome participant complete clearance was reported in most of the included studies and revealed varying rates for the different interventions. Diclofenac/HA showed rather low clearance rates of 35% in the meta-analysis of single-armed studies and was also inferior in a direct comparison to imiquimod, which is in line with the results from previous RCTs [27,28]. Participant complete clearance rates for ALA-PDT strongly contrast with findings from pivotal RCTs in which at least twice as many patients achieved complete clearance of their AKs [29,30]. In contrast, complete clearance for IMB in this analysis was almost twice as high as reported by a pivotal phase III RCT comparing IMB to placebo [31]. However, cross-trial comparisons need to be interpreted cautiously. A possible explanation for the discrepant results may lie in the differences of the methodological approaches and the study populations. RCTs are mostly designed to minimise the risk of bias, for instance, through blinding of the participants or the outcome assessor as well as through counteracting selection bias. In contrast, phase IV trials do not underlie such a stringent protocol, as the medications have already been licensed. Furthermore, the blinding of participants is rarely possible. Nevertheless, discordant results from pivotal and post-marketing surveillance trials can pose a major challenge for prescribing clinicians and regulatory authorities, as has recently been the case for IMB. Furthermore, it remains unclear whether results from sound RCTs or well-conducted phase IV trials are more reliable and generalizable.

Surprisingly, none of the studies identified in our systematic literature research investigated 5-FU, although a recent head-to-head trial showed that 5-FU was the most effective as well as the most cost-effective intervention for managing AK in comparison to PDT, imiquimod 5% cream, and IMB 0.015% gel [32,33,34]. In our study, the number of withdrawals due to AEs was slightly higher among the patients who had treated their AK with diclofenac/HA in comparison to the remaining treatment regimens. This may be explained by the fact that diclofenac/HA has to be applied twice daily for 60 to 90 days according to the summary of product characteristics, while IMB only has to be self-applied for three consecutive days. In contrast, PDT represents a hospital-based approach, which is mostly performed in only one treatment session. Therefore, withdrawals due to AEs are rarely reported.

Data on patients with recurrent lesions were only reported in three studies. Values varied from 80–90% for imiquimod 5% cream and diclofenac/HA in the LEIDA studies, respectively [17]. In contrast, only 10% to 30% of patients reported recurrent lesions for IMB and PDT, respectively. However, the time of the outcome assessment differed across these studies and was longest in the LEIDA studies with a follow-up of 36 months. This difference might explain the higher rates in these studies. It is conceivable that the recurrence rates of the other treatment regimens may increase over time as well, and longer follow-up data are urgently needed to allow for comparisons of AK treatments over a longer period.

The interpretation of the trials included in this study was challenged by the presence of multiple interventions, comparisons, and study designs. The definition of phase IV or post-marketing surveillance trials is rather vague. They encompass any study conducted within the conditions of the approved summary of product characteristics or under normal conditions of use [8,9,10]. This covers both interventional clinical trials (phase IV sensu strictu) and non-interventional studies, which is also displayed by the various study designs of the records included in this analysis. Some studies used a randomized or non-randomized controlled approach and investigated different substances in head-to-head trials, whereas others were only interested in one intervention and refrained from a control group. This heterogeneity arising due to different study designs as well as interventions is a major limitation and challenges the interpretation of our results. In any case, future post-marketing surveillance trials should minimise potential sources of bias.

Nevertheless, our study is among the first to dissect data from phase IV studies as a proxy for the treatment of AK in a real-world context. Due to the high number of more than 4000 patients included in this analysis, we believe that the findings are generalizable and will help dermatologists to judge and compare the results of previous pivotal trials, particularly in terms of long-term efficacy and safety.

## Figures and Tables

**Table 1:** Baseline characteristics of the included studies.

Study	Design	Localization of AK	Intervention	Control
Berlin 2008	prospective, randomized, multicentre, open-label, phase IV study	forehead scalp hands	cryosurgery with a single freeze-thaw cycle and 4–10 s freeze time, followed by diclofenac sodium 3% in hyaluronic acid gel 2.5% 15 days after cryosurgery for 90 days (*n* = 368)	cryosurgery with a single freeze-thaw cycle and 4–10 s freeze time (*n* = 346)
Dirschka 2010	open-label, randomized, multicentre phase IV study	face scalp	diclofenac sodium 3% in hyaluronic acid gel 2.5% twice daily for 3 months (*n* = 65)	uncontrolled
Gollnick 2019	2 randomized, active-controlled, open-label, multicentre, multinational, phase IV studies (NCT00777127/NCT01453179)	face scalp	application of imiquimod 5% cream 3 nights per week for 4 weeks followed by a 4 week treatment pause. If the lesions had not cleared, the patient received a second 4 week course of treatment (*n* = 242)	application of diclofenac sodium 3% in hyaluronic acid gel 2.5% twice daily for 12 weeks followed by an 8-week off-treatment phase (*n* = 237)
Kim 2018	open-label, multicentre, parallel-group, phase IV study	face scalp extremities	face/scalp: application of ingenol mebutate gel 0.015% for 3 consecutive days (*n* = 67) trunk/extremities: application of ingenol mebutate gel 0.05% for 2 consecutive days (*n* = 10)	uncontrolled
Nelson 2009	open-label, single-arm, multicentre, phase IV study	forehead central face scalp	application of diclofenac sodium 3% in 2.5% hyaluronic acid gel for 90 days (*n* = 76)	uncontrolled
Reinhold 2016	multicentre, prospective, non-interventional study	hands forearm wrist other	application of 0.5% 5-fluorouracil solution in combination with 10% salicylic acid once a day, no fixed treatment period was defined due to the non-interventional character of the study (*n* = 649)	uncontrolled
Reinhold 2018	multicentre, prospective observational case-only study	face scalp	application of 5-ALA patch for 4 h. Subsequently, the patch was removed and the lesions were illuminated with red LED light (630 ± 3 nm, 37 J/cm^2^). (*n* = 386)	uncontrolled
Stockfleth 2018	open-label, multicentre, randomized, active-controlled, head-to-head, phase IV study	face scalp	application of ingenol mebutate gel 0.015% once daily for 3 consecutive days (*n* = 255)	application of diclofenac sodium 3% in hyaluronic acid gel 2.5% twice daily for 90 days (*n* = 247)
Szeimies 2015	open-label, multicentre, non-interventional study	head face arms/hands legs trunk	application of 0.5% 5-fluorouracil solution in combination with 10% salicylic acid once daily (*n* = 1051)	uncontrolled
Tschen 2006	open-label, multicentre, phase IV study	face scalp	application of topical ALA solution. After an incubation period of 14–18 h, the ALA-treated lesions were rinsed gently with water, patted dry and exposed to 10 J/cm^−2^ of visible blue light (417 ± 4 nm peak) delivered at 10 mW/cm^2^, persisting lesions were re-treated at month 2 (*n* = 110)	uncontrolled

AK: actinic keratosis; ALA: aminolevulinate.

**Table 2:** Summary of the primary and secondary outcomes.

Study	Intervention	Primary Outcomes				Secondary Outcomes	
		Participant Complete Clearance	Lesion-Specific Clearance	Mean Lesion Complete Clearance Per Patient	Recurrence Rate	Patient Satisfaction	Withdrawal due to Adverse Events
Controlled Trials							
Berlin 2008	Cryotherapy + diclofenac/HA	42.4% (156/368)	n.r.	8.9 to 1.1 (88% decrease)	n.r.	n.r.	8.4% (31/368)
	Cryotherapy	25.7% (89/346)	n.r.	8.2 to 2.7 (67% decrease)	n.r.	n.r.	1.2% (4/346)
	Effect estimate	RR 1.65 95% CI 1.33–2.04	n.r.	n.e.	n.r.	n.r.	RR 7.29 95% CI 2.60–20.42
LEIDA 1 + LEIDA 2 (Gollnick 2019)	Diclofenac/HA	35.4% (84/237)	n.r.	n.r.	90.7% (215/237)	n.r.	n.r.
	Imiquimod 5%	52.1% (126/242)	n.r.	n.r.	82.6% (200/242)	n.r.	n.r.
	Effect estimate	RR 0.68 95% CI 0.55–0.84	n.r.	n.r.	RR 1.10 95% CI 1.02–1.18	n.r.	n.r.
Stockfleth 2018	IMB 0.015%	45.1% (115/255)	n.r.	69.5% decrease	n.r.	No specific values reported	2.7% (7/255)
	Diclofenac/HA	23.5% (58/247)	n.r.	57.7% decrease	n.r.		6.1% (15/247)
	Effect estimate	RR 1.92 95% CI 1.48–2.50	n.r.	n.e.	n.r.	n.r.	RR 0.45 95% CI 0.19–1.09
Single-Armed Trials							
Dirschka 2010	Diclofenac/HA	23.1% (15/65)	n.r.	n.r.	n.r.	n.r.	n.r.
Nelson 2009	Diclofenac/HA	48.7% (37/76)	n.r.	8.4 to 0.8 (90.5% decrease)	n.r.	n.r.	3.9% (3/76) *
Reinhold 2016	5-FU/SA	n.r.	n.r.	from 3.9 ± 2.8 to 0.3 ± 1.1	n.r.	n.r.	1.4% (9/649)
Szeimies 2015	5-FU/SA	n.r.	n.r.	4.1 to 1.2 (69.7% decrease)	n.r.	Patients were satisfied	2.3% (24/1051)
Reinhold 2018	ALA-PDT (patch)	n.r.	84.3% (1160/1376)	n.r.	n.r.	n.r.	n.r.
Tschen 2006	ALA-PDT	33.6% (37/110)	61.2% (458/748)	n.r.	23.5% (162/688)	n.r.	1.8% (2/110)
Kim 2018	IMB 0.015%	76.6% (49/64)	n.r.	88.3% decrease ±37.3%	10.6% (5/47)	n.r.	1.3% (1/77)
	IMB 0.05%	88.9% (8/9)	n.r.		28.6% (2/7)	n.r.	

* Data was obtained from Nelson 2004. RR: relative risks; HA: hyaluronic acid; IMB: ingenol mebutate; FU: fluorouracil; SA: salicylic acid; ALA: aminolevulinate; PDT: photodynamic therapy.

**Table 3:** Balance sheet comparing the interventions and outcomes at a glance.

	Cryotherapy	Cryotherapy + Diclofenac/HA	Diclofenac/HA	Imiquimod 5%	IMB	5-FU/SA	ALA-PDT
Participant Complete Clearance	25.7%	42.4%	23.5%–48.7%	52.1%	0.015%: 45.1%–76.6% 0.05%: 88.9%	n.r.	33.6%
Recurrence Rate	n.r.	n.r.	90.7%	82.6%	0.015: 10.6% 0.05%: 28.6%	n.r.	23.5%
Withdrawal due to Adverse Events	1.2%	8.4%	3.9%–6.1%	n.r.	0.015%: 2.7% 0.05%/0.015%: 1.3%	1.4%–2.3%	1.8%

## Supplementary Materials

The following are available online at https://www.mdpi.com/2077-0383/9/7/2253/s1, Figure S1: (a) Risk ratio for a participant to have all AK (100%) cleared for diclofenac/HA, (b) risk ratio for lesions to be cleared for the intervention ALA-PDT, and (c) risk ratio for a participant to withdraw from the study due to adverse events when treated with 5-fluorouracil solution 0.5% in combination with 10% salicylic acid (5-FU/SA). The forest plots show pooled data from single-armed studies (random-effects analysis). The diamond represents the exact estimate from the study. The width of the line extending from each diamond represents the 95% confidence interval (CI); Table S1: Search strategy in the databases.

## Abbreviations

5-FU/SA	5-fluorouracil solution 0.5% in combination with 10% salicylic acid
AK	actinic keratosis
ALA	5-aminolevulinic acid
CI	confidence interval
cSCC	squamous cell carcinoma of the skin
Diclofenac/HA	diclofenac sodium 3% in hyaluronic acid gel 2.5%
EMA	European Medicines Agency
HA	hyaluronic acid
IMB	ingenol mebutate
MAL	methyl-aminolevulinic acid
n.a.	not assessible
n.e.	not estimable
n.r.	not reported
PDT	photodynamic therapy
RCT	randomized controlled trial
RR	risk ratio
YAG	yttrium aluminium garnet
