# Supplementary material for: A Systematic Review and Meta-Analysis of Interventions for Actinic Keratosis from Post-Marketing Surveillance Trials
Source: J Clin Med. 2020 Jul 15;9(7):2253. doi: 10.3390/jcm9072253 (PMC7408895; doi:10.3390/jcm9072253)

Supplementary file

Supplementary Table S1: Search strategy in the databases.

**Ovid MEDLINE(R) and Epub Ahead of Print, In-Process & Other Non-Indexed Citations, Daily and Versions(R) 1946 to March 24, 2020**<br>**N=23**
1. actinic keratosis.mp. or exp Keratosis, Actinic/
2. solar keratosis.mp.
3. senile keratosis.mp.
4. field change.mp.
5. actinically damaged field.mp.
6. exp Precancerous Conditions/ or field-cancerized.mp.
7. 1 or 2 or 3 or 4 or 5 or 6
8. excision.mp.
9. exp Biopsy/ or shave.mp.
10. curettage.mp. or exp CURETTAGE/
11. laser.mp. or exp Lasers/
12. cryotherapy.mp. or exp CRYOTHERAPY/
13. cryosurgery.mp. or CRYOSURGERY/
14. cryopeel*.mp.
15. fluorouracil.mp. or exp FLUOROURACIL/
16. fluorouracil derivative.mp.
17. efudix.mp.
18. actikerall.mp.
19. 5-FU.mp.
20. exp Aminoquinolines/ or imiquimod.mp.
21. aldara.mp.
22. zyclara.mp.
23. exp Diterpenes/ or ingenol mebutate.mp.
24. picato.mp.
25. diclofenac.mp. or exp DICLOFENAC/
26. solaraze.mp.
27. solacutan.mp. [mp=title, abstract, original title, name of substance word, subject heading word, floating sub-heading word, keyword heading word, organism supplementary concept word, protocol supplementary concept word, rare disease supplementary concept word, unique identifier, synonyms]
28. photodynamic therapy.mp. or exp Photochemotherapy/
29. exp Aminolevulinic Acid/ or aminolevulinate.mp. or exp Photosensitizing Agents/
30. methyl aminolevulinic acid.mp.
31. MAL.mp.
32. ALA.mp.
33. BF-200 ALA.mp.
34. Ameluz.mp.
35. Alacare.mp.
36. Metvix.mp.

37. Luxerm.mp. [mp=title, abstract, original title, name of substance word, subject heading word, floating sub-heading word, keyword heading word, organism supplementary concept word, protocol supplementary concept word, rare disease supplementary concept word, unique identifier, synonyms]<br>38. skin surgery.mp. or exp Dermatologic Surgical Procedures/<br>39. phase 4.mp.<br>40. phase IV.mp.<br>41. exp Product Surveillance, Postmarketing/ or post-marketing surveillance.mp.<br>42. non-interventional study.mp.<br>43. 39 or 40 or 41 or 42<br>44. 8 or 9 or 10 or 11 or 12 or 13 or 14 or 15 or 16 or 17 or 18 or 19 or 20 or 21 or 22 or 23 or 24 or 25 or 26 or 27 or 28 or 29 or 30 or 31 or 32 or 33 or 34 or 35 or 36 or 37 or 38<br>45. 7 and 43 and 44
**Embase 1974 to 2020 March 24**<br>**N=46**
1. actinic keratosis.mp. or actinic keratosis/<br>2. solar keratosis.mp.<br>3. senile keratosis.mp.<br>4. field change.mp.<br>5. actinically damaged field.mp.<br>6. field-cancerized.mp. or exp precancer/<br>7. 1 or 2 or 3 or 4 or 5 or 6<br>8. excision.mp. or exp excision/ or exp wide excision/ or exp local excision/<br>9. exp skin biopsy/ or shave.mp.<br>10. curettage.mp.<br>11. laser.mp. or exp carbon dioxide laser/ or neodymium YAG laser/ or exp erbium YAG laser/ or exp excimer laser/ or laser/<br>12. cryotherapy.mp. or exp cryotherapy/<br>13. exp cryosurgery/ or cryosurgery.mp.<br>14. cryopeel*.mp. or exp skin surgery/<br>15. exp fluorouracil/ or exp fluorouracil plus salicylic acid/ or exp fluorouracil derivative/ or fluorouracil.mp.<br>16. efudix.mp.<br>17. actikerall.mp.<br>18. 5-FU.mp.<br>19. exp imiquimod/ or imiquimod.mp.<br>20. aldara.mp.<br>21. zyclara.mp.<br>22. ingenol mebutate.mp. or exp ingenol mebutate/<br>23. picato.mp.<br>24. diclofenac.mp. or exp diclofenac/ or exp diclofenac derivative/<br>25. solaraze.mp.<br>26. solacutan.mp. [mp=title, abstract, heading word, drug trade name, original title, device manufacturer, drug manufacturer, device trade name, keyword, floating subheading word, candidate term word]<br>27. photodynamic therapy.mp. or exp photodynamic therapy/<br>28. exp phototherapy/

29. aminolevulinate.mp. or exp aminolevulinic acid/<br>30. exp aminolevulinic acid methyl ester/ or methyl aminolevulinic acid.mp.<br>31. MAL.mp.<br>32. exp nanoemulsion/ or exp photosensitizing agent/ or BF-200 ALA.mp.<br>33. Ameluz.mp.<br>34. Alacare.mp.<br>35. Metvix.mp.<br>36. Luxerm.mp. [mp=title, abstract, heading word, drug trade name, original title, device manufacturer, drug manufacturer, device trade name, keyword, floating subheading word, candidate term word]<br>37. exp curettage/<br>38. ALA.mp.<br>39. 8 or 9 or 10 or 11 or 12 or 13 or 14 or 15 or 16 or 17 or 18 or 19 or 20 or 21 or 22 or 23 or 24 or 25 or 26 or 27 or 28 or 29 or 30 or 31 or 32 or 33 or 34 or 35 or 36 or 37 or 38<br>40. phase 4.mp.<br>41. exp phase 4 clinical trial/ or phase IV.mp.<br>42. non-interventional.mp.<br>43. post-marketing surveillance.mp. or exp postmarketing surveillance/<br>44. 40 or 41 or 42 or 43<br>45. 7 and 39 and 44
**Cochrane Library Central**<br>**N=141 trials**
#1 MeSH descriptor: [Keratosis, Actinic] explode all trees<br>#2 actinic keratos*<br>#3 solar keratos*<br>#4 senile keratos*<br>#5 field cancerization<br>#6 precancerous lesion<br>#7 #1 or #2 or #3 or #4 or #5 or #6<br>#8 Phase 4<br>#9 Phase IV<br>#10 post-marketing surveillance<br>#11 postmarketing surveillance<br>#12 non-interventional<br>#13 #8 or #9 or #10 or #11 or #12<br>#14 #7 AND #13

Supplementary figure 1

(a) Participant complete clearance

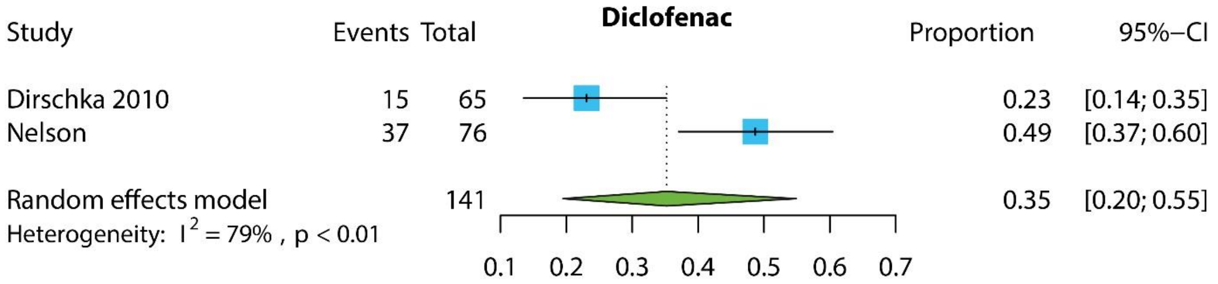

(b) Lesion complete clearance

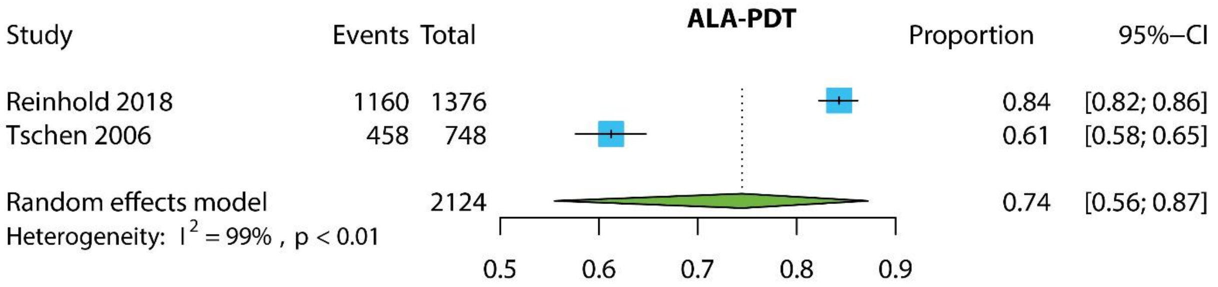

(c) Withdrawal due to AE

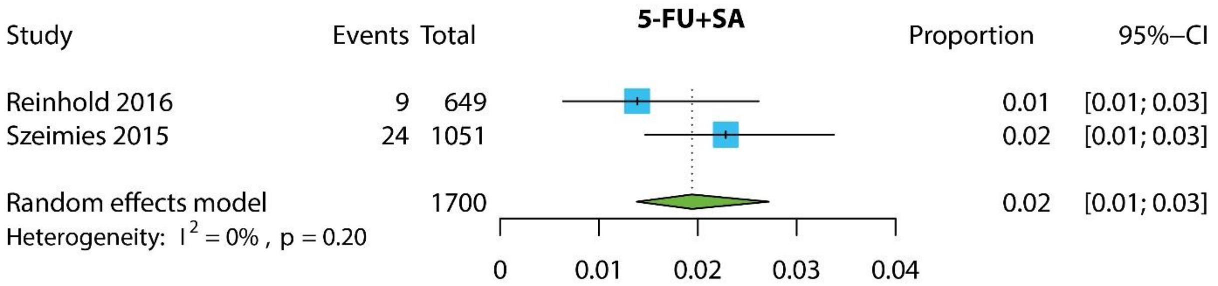

Supplement: Supplementary file 1 [file jcm-09-02253-s001.pdf]
